# F1012-2 Induced ROS-Mediated DNA Damage Response through Activation of MAPK Pathway in Triple-Negative Breast Cancer

**DOI:** 10.1155/2021/6650045

**Published:** 2021-06-01

**Authors:** Lingjie Dai, Shasha Tian, Jinyao Zhang, Mengyuan Lu, Jingchao Zhu, Huajun Zhao

**Affiliations:** School of Pharmaceutical Sciences, Zhejiang Chinese Medical University, Hangzhou, Zhejiang 311402, China

## Abstract

We have previously reported that F1012-2, a sesquiterpene lactone isolated from the Chinese herbal medicine *Eupatorium lindleyanum* DC., exhibits strong effects against Triple Negative Breast Cancer (TNBC). In this study, we found F1012-2 effectively inhibited cell migration and invasion detected by wound healing and transwell assays. In order to elucidate the potential mechanisms of F1012-2, we further studied its effect on DNA damage in TNBC cell lines. Using single cell gel electrophoresis (comet assay), immunofluorescence, and western blotting assays, we found that F1012-2 treatment induced significant DNA strand breaks and *γ*-H2AX activation. Moreover, exposure to F1012-2 led to overproduction of reactive oxygen species (ROS). NAC treatment completely eliminated ROS, which may be due to the interaction between NAC and F1012-2. A further study of the molecular mechanisms demonstrated that the MAPK signaling pathway participated in the anti-TNBC effect of F1012-2. Pretreatment with specific inhibitors targeting JNK (SP600125) and ERK (PD98059) could rescue the decrease in cell viability and inhibit expressions of JNK and ERK phosphorylation, but SB203580 had no effects. Finally, in the acute toxicity experiment, there were no obvious symptoms of poisoning in the F1012-2 treatment group. An *in vivo* study demonstrated that F1012-2 significantly suppressed the tumor growth and induced DNA damage. In conclusion, the activity of F1012-2-induced DNA damage in TNBC was found *in vivo* and *in vitro*, which might trigger the MAPK pathway through ROS accumulation. These results indicate that F1012-2 may be an effective anti-TNBC therapeutic agent.

## 1. Introduction

Breast cancer is a common disease in women and ranks the first in the incidence of female cancer at 30% and the second in the mortality rate at 15% [1]. Triple negative breast cancer (TNBC) is considered to be one of the most drug-resistant, metastatic, and difficult-to-treat breast cancer subtypes. It is characterized by the lack of estrogen receptor (ER), progesterone receptor (PR), and human epidermal growth factor receptor 2 (HER2) [2, 3]. Due to the lack of molecular targets, no molecularly targeted therapeutic agents have been clinically approved for TNBC, and conventional treatments (such as radiotherapy and chemotherapy) are still the main means of systemic treatment [4, 5]. Therefore, new therapeutic strategies for TNBC are urgently needed. Natural products are becoming more and more popular due to their structural diversity, higher multitarget activity, less toxicity, and side effects [6]. A variety of natural products have entered the market or clinical research stage of cancer treatment, and research on the mechanisms is also increasing [7]. Therefore, natural products will play an important role for new drugs in the future [8].

*Eupatorium lindleyanum* DC. belongs to the family Asteraceae and is mainly used to treat cough, phlegm, tonsillitis, chronic bronchitis, bronchitis, hypertension, etc. [9, 10]. In recent years, it has been found to be rich in active ingredients, mainly including sesquiterpenes, flavonoids, triterpenes, volatile oils, and organic acids. Furthermore, it also shows important medicinal values in antitumor and antivirus. F1012-2 is an active fraction of new sesquiterpene lactone isolated from *Eupatorium* lindleyanum DC. Our previous studies have found that it has potent antihuman breast cancer activity [11], but its exact mechanisms have not been yet clear.

Reactive oxygen species (ROS) are by-products of oxidative metabolism and play a dual role in cancer development [12]. Normal levels of ROS regulate cell proliferation, angiogenesis, and invasion, while excessive ROS can cause oxidative stress, thus inducing DNA damage and triggering various cellular responses [13, 14]. The mitogen-activated protein kinase (MAPK) signaling pathway is involved in many physiological processes such as cell growth, development, division, and differentiation [15, 16]. Recent studies have emphasized that ROS can activate the Ras/MAPK pathway [17, 18]. Extracellular signal-regulated kinase (ERK), c-Jun N-terminal kinase (JNK), and p38 MAPK are all members of the MAPK family. They have different cellular functions [19]. Generally, the activation of ERK can promote cell proliferation and growth, but the continuous activation of ERK by extremely high ROS can induce tumor cell apoptosis [20]. JNK1 and JNK2 in the JNK family are widely expressed throughout the human body and can phosphorylate a variety of proteins such as c-Jun [21]. P38 is a stress-activated protein kinase, and excessive ROS will cause the activation time of JNK/p38 to be prolonged, thereby inhibiting the proliferation of tumor cells.

Here, we show that F1012-2 inhibits TNBC cell migration and invasion and induces overproduction of ROS, which leads to DNA damage. Further studies showed that F1012-2 activated the MAPK signaling pathway. In brief, our data suggests that F1012-2 may be a new and effective drug for the treatment of TNBC. The study of the anti-TNBC mechanism of F1012-2 will help to develop better therapeutic strategies against TNBC.

## 2. Materials and Methods

### 2.1. Cell Culture and Reagents

Human breast cancer cell lines MDA-MB-231 and MDA-MB-468 were purchased from the Shanghai Institute of Materia Medica, Chinese Academy of Sciences (Shanghai, China). The cells were cultured in Dulbecco's modified Eagle's medium (DMEM) supplemented with 10% fetal bovine serum (FBS) and 1% penicillin-streptomycin (both from Gibco, USA) at 37°C with 5% CO_2_. The sesquiterpene lactone active fraction F1012-2 was provided by Dr. Yang Bo (Zhejiang Chinese Medical University, Hangzhou, China). PD98059, SP600125, and SB203580 were purchased from Shanghai University Biotechnology Company.

### 2.2. Cell Viability Assay

Cell growth inhibition was measured by MTT assay. The cells were seeded in a 96-well plate (3 × 10^3^ cells per well) to allow attachment and incubated overnight in a medium containing 10% FBS at 37°C. Expose to different drugs and cultivate for 48 hours. Add MTT solution (5 mg/ml) to each well, and add DMSO after 4 hours to dissolve the formazan crystals. Absorbance was measured at 570 nm with a Cell Imaging Multi-Mode Reader (BioTek, CA, USA).

### 2.3. Wound Healing Assay

The cells were seeded in a 24-well plate (2 × 10^5^ cells per well). When the cells covered the bottom of the plate, a 200 *μ*l pipette tip was used to make cell scratches, washed with PBS three times, and incubated with different concentrations of F1012-2. Then, pictures were taken and recorded at 0 and 24 hours.

### 2.4. Transwell Assay

Take log phase cells, and prepare a cell suspension of 5 × 10^5^ cells/ml with serum-free medium containing 0.5% BSA. Remove the chamber, add 100 *μ*l of cell suspension to the upper chamber (with or without Matrigel), and add 600 *μ*l of complete medium containing 10% FBS to the lower chamber. Place the cells in a cell culture incubator at 37°C, with 5% CO_2_ and saturated humidity for 24 h. After 24 hours, the culture was terminated, the cells were taken out of the incubator, and the upper chamber liquid was discarded. Then, cells were fixed with methanol for 5 minutes, washed 3 times with PBS, and stained with hematoxylin and eosin for 5 minutes each. Observe under an upright microscope, take pictures, and calculate the relative migration rate of cells.

### 2.5. DNA Damage (Strand Breakage)-Comet Assay

The cells were exposed to F1012-2 for 24 hours at doses of 0, 4, and 8 *μ*g/ml. Collect the cells, and mix the cells with 0.6% low melting point agarose. Spread the mixture on a comet slide, place it in a dark environment at 4°C for 30 minutes, and then immerse it in freshly prepared cold cracking solution. Finally, the slides were electrophoresed in a horizontal electrophoresis apparatus at a speed of 1.0 V/cm and stained with PI in the dark. Use the fluorescence microscope to acquire images.

### 2.6. Immunofluorescence Staining

After being exposed to 8 *μ*g/ml F1012-2 for 24 hours, the cells were fixed with 4% paraformaldehyde and permeabilized in 0.5% Triton X-100. After blocking with 2% BSA, the cells and *γ*-H2AX antibody were incubated overnight. The cells were incubated with a secondary antibody for 1 hour on the second day. Finally, DAPI staining was performed for 10 min and direct photography.

### 2.7. Measurement of Intracellular ROS

Antioxidant fluorescent probes (Beyotime, Shanghai, China) were used to detect intracellular ROS levels. Briefly, the cells were pretreated with or without NAC (5 mM) for 2 hours and then treated with F1012-2 for 24 hours. The blank medium containing DCFH-DA probe was added to make the probe fully interact with the cells for 15 min, and the uncombined dyes were washed away with PBS. The fluorescence intensity was detected by flow cytometry.

### 2.8. Western Blotting Analysis

Lyse cells with RIPA buffer, which contains protease inhibitors and phosphatase inhibitors. Then, the equivalent protein mass was separated by SDS-PAGE and transferred to PVDF membrane. Incubate with 5% skim milk to block nonspecific binding sites, then incubate with specific primary antibody overnight, and detect a primary antibody with a secondary antibody. The following primary antibodies were used: *γ*-H2AX, ERK, p-ERK, JNK1, JNK2, p-JNK, p-c-Jun, p38, and p-p38. *β*-Tubulin and GAPDH were used as an internal control. Use ECL (Bio-Rad, USA) for chemiluminescence detection.

### 2.9. Acute Toxicity Study

Twenty ICR mice were divided into two groups, ten mice in each group, with 5 males and 5 females. Before administration, all mice were fasted for 12 hours and intragastrically administered at the maximum dose of 2000 mg/kg once within a day (each time, 0.4 ml/10 g). Observe the animal reaction within 4 hours and whether there are abnormal calls, drooling, tearing, dyspnea, diarrhea, and constipation. The body weight was recorded every week, and the mice were sacrificed 14 days later, and the organs were visually observed for abnormalities.

### 2.10. In Vivo Animal Studies

The transplanted tumor model was established by subcutaneous injection of 2 × 10^6^ MDA-MB-231 cells into 4-week-old female BALB/c mice. When the tumors were visible to the naked eye (at least 50 mm^3^), the control group received intraperitoneal injection of vehicle control every other day and the treatment group received 15 mg/kg F1012-2 intraperitoneal injection every other day. Measure the tumor size every other day, and use the formula to calculate the tumor volume: volume = (width^2^ × length)/2. Take out the tumor for histological analysis after 20 days. Animal experiments were approved by the Institutional Animal Care and Use Committee of Zhejiang Chinese Medical University.

### 2.11. Immunohistochemical Assay

The formalin-fixed and paraffin-embedded tumor tissue samples were cut into 5-micron-thick sections. After the slices are baked, deparaffinized, and rehydrated, they are incubated in hydrogen peroxide for 10 minutes. Subsequently, the slides were incubated with the *γ*-H2AX primary antibody at 4°C. The next day, the slides were incubated with the secondary antibody at 37°C. Finally, the tumor tissue sections were stained with DAB, counterstained with hematoxylin, dehydrated, and covered with a cover glass.

### 2.12. Statistical Analysis

All data are expressed as the mean ± SD. Student's *t*-test was used to analyze statistical significance. The standard of statistical significance is ^∗^*p* < 0.05, ^∗∗^*p* < 0.01, and ^∗∗∗^*p* < 0.001.

## 3. Results

### 3.1. F1012-2 Inhibits MDA-MB-231 and MDA-MB-468 Cell Migration and Invasion

**Figure 1:**
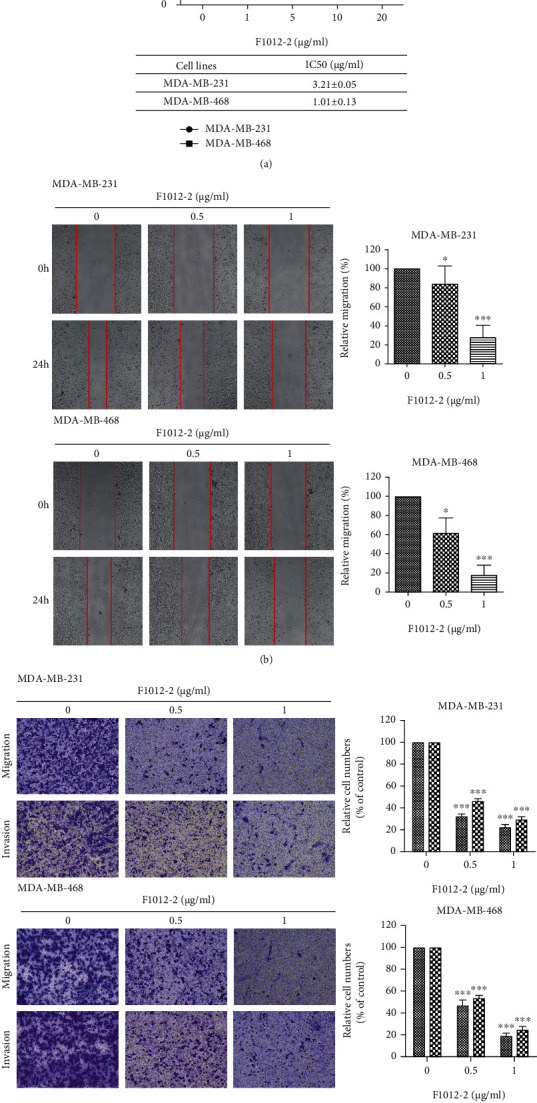
F1012-2 inhibits MDA-MB-231 and MDA-MB-468 cell migration and invasion. (a) The cells were incubated with F1012-2 for 48 h, and then, the cell viability was measured by MTT assay. (b) After 24 hours of treatment with F1012-2, the cell migration was detected by wound healing. (c) The cell migration and invasion were detected by transwell. ^∗^*p* < 0.05, ^∗∗^*p* < 0.01, and ^∗∗∗^*p* < 0.001.

In order to evaluate the effect of F1012-2 on cell migration and invasion, we determined through MTT experiment that the IC_50_ of MDA-MB-231 and MDA-MB-468 cells are 3.21 ± 0.05 *μ*g/ml and 1.01 ± 0.13 *μ*g/ml, respectively (Figure 1(a)). Cell migration and invasion experiments were carried out at concentrations of 0.5 and 1 *μ*g/ml with less significant inhibition of cell proliferation. In the wound healing and transwell experiments, F1012-2 treatment can make the cell migration slower and the ability of cells to pass through the chamber and Matrigel matrix was reduced (Figures 1(b) and 1(c)). In summary, these results indicate that F1012-2 can effectively inhibit cell migration and invasion.

### 3.2. F1012-2 Induces DNA Damage in MDA-MB-231 and MDA-MB-468 Cells

**Figure 2:**
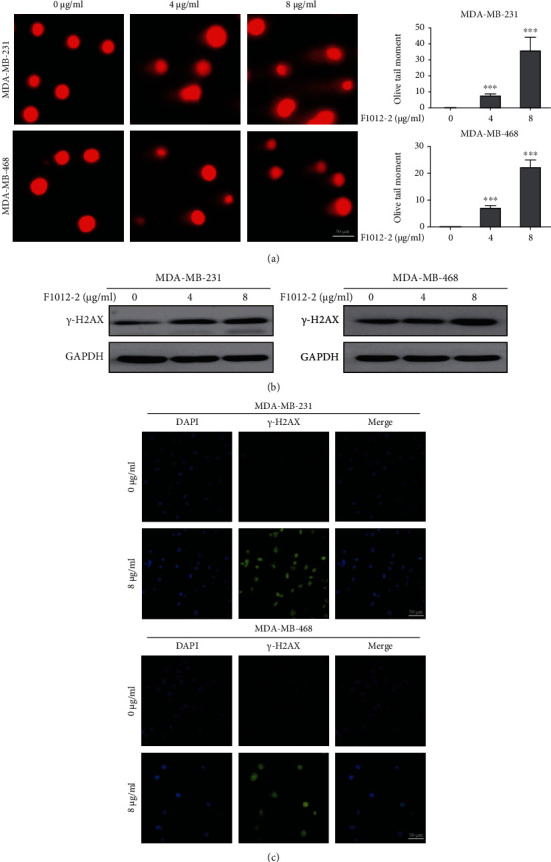
F1012-2 induces DNA damage in MDA-MB-231 and MDA-MB-468 cells. (a) The cells were treated with F1012-2 (0, 4, and 8 *μ*g/ml) for 24 hours, and then, DNA damage (strand breaks) was determined with comet assay. (b) After 24 hours of treatment with F1012-2, total cellular protein was extracted from two cells, and the levels of *γ*-H2AX were detected by western blotting. (c) The representative images of *γ*-H2AX immunofluorescence staining in two cells treated with F1012-2 for 24 hours. Scale bars = 50 *μ*m.

To evaluate whether F1012-2 induced DNA damage, the cells were incubated with F1012-2 (0, 4 and 8 *μ*g/ml) and analyzed by comet assay. The results indicated that F1012-2 led to DNA strand breaks in two cells in a dose-dependent manner (Figure 2(a)). An increase in double strand breaks was shown by significantly increased levels of *γ*-H2AX (Ser-139), an indicator of double-stranded DNA breaks [22, 23], detected by western blotting and immunofluorescence analysis (Figure 2(b) - 2(c)). These results indicated that F1012-2 treatment induced DNA damage in TNBC cells.

### 3.3. F1012-2 Induces ROS Overproduction in MDA-MB-231 and MDA-MB-468 Cells

**Figure 3:**
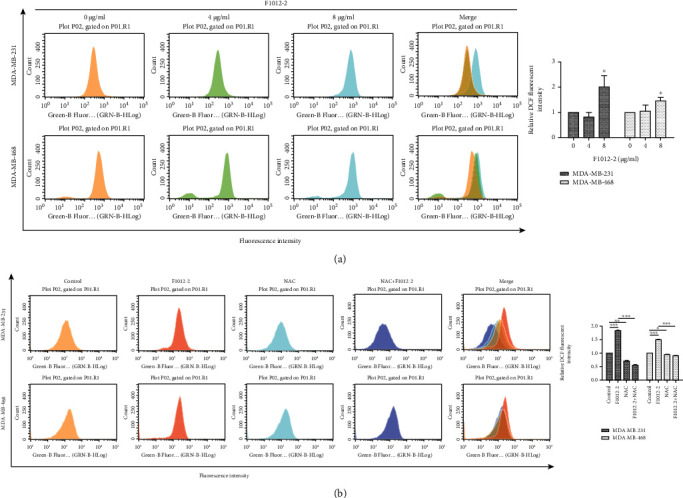
F1012-2 induces ROS overproduction in MDA-MB-231 and MDA-MB-468 cells. (a) After treatment with F1012-2, the cells were stained with DCFH-DA, and the fluorescence was analyzed by flow cytometry. (b) The cells were pretreated with NAC (5 mM) for 2 h and then incubated with F1012-2. Flow cytometry was used to detect ROS. ^∗^*p* < 0.05, ^∗∗^*p* < 0.01, and ^∗∗∗^*p* < 0.001.

It is well recognized that the accumulation of ROS leads to a significant increase in oxidative DNA damage [24]. To detect ROS production in two cells, we examined with DCFH-DA. F1012-2 treatment significantly increased the levels of intracellular ROS in a dose-dependent manner in the two cell lines compared to the control (Figure 3(a)). These results indicated that ROS accumulation was involved in F1012-2-induced DNA damage in TNBC cells. In order to further evaluate whether F1012-2 induces an increase in ROS levels, we pretreated with antioxidant N-acetyl-L-cysteine (NAC) for 2 hours and found that ROS was completely eliminated by NAC (Figure 3(b)). Recent studies have shown that sesquiterpene lactones can bind to nucleophilic thiol compounds, including L-cysteine [25], which may explain this phenomenon.

### 3.4. F1012-2 Activates MAPK Pathway in MDA-MB-231 and MDA-MB-468 Cells

When cell DNA is damaged, ATM and other proteins first recognize the damage and then transmit the signal to the downstream MAPK pathway. The activation of the MAPK pathway can cause the cell cycle arrest in order to facilitate the repair of DNA or initiate the apoptosis mechanism when DNA damage cannot be repaired. In order to elucidate the possible mechanism by which F1012-2 induces DNA damage in TNBC, we evaluated its effect on MAPK signaling pathways.

**Figure 4:**
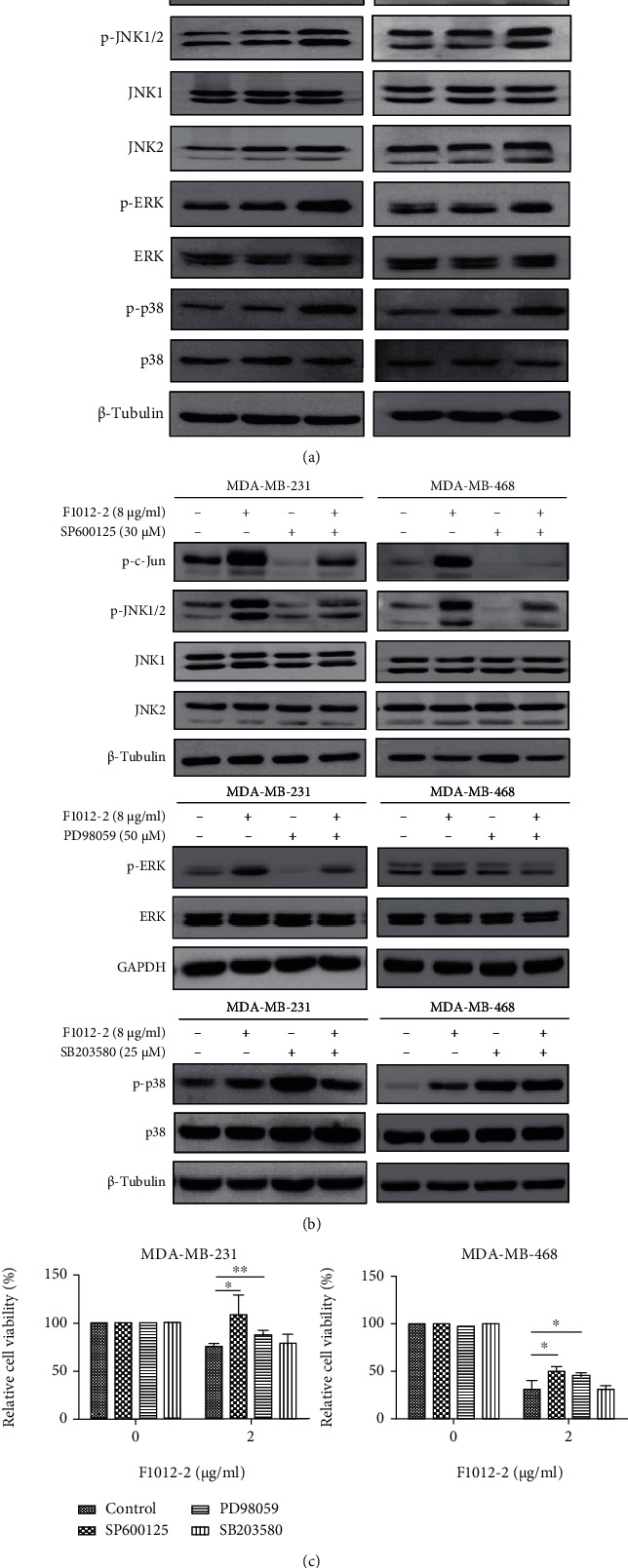
F1012-2 activates the MAPK pathway in MDA-MB-231 and MDA-MB-468 cells. (a) Total cell proteins were extracted after treatment with F1012-2, and protein levels were detected by western blot. (b) The cells were pretreated with SP600125, PD98059, and SB203580 for 30 minutes and then incubated with F1012-2. Western blotting was used to detect protein expression. (c) The cells were pretreated with inhibitors; then, the cell viability was measured by the MTT assay. ^∗^*p* < 0.05 and ^∗∗^*p* < 0.01.

Previously, we had shown that the p38-MAPK signaling pathway is partly involved in F1012-2-induced TNBC cell apoptosis [11]. In this study, we found F1012-2 significantly increased the phosphorylation levels of JNK, c-Jun (the substrate of JNK), p38, and ERK, but their total protein levels did not change significantly (Figure 4(a)). To investigate the role of MAPK activation in F1012-2-induced cytotoxicity, cells were pretreated with JNK (SP600125), P38 (SB203580), and ERK (PD98059) inhibitors. As shown in Figures 4(b) and 4(c), pretreatment with SP600125 and PD98059 inhibitors could rescue the decrease in cell viability caused by F1012-2, while SB203580 has no significant effect. A further study confirmed that SP600125 and PD98059, respectively, inhibited the phosphorylation levels of JNK and ERK proteins, but SB203580 had no effect detected by western blotting (Figure 4(b)). These data indicated that the JNK and ERK pathways mainly contributed to F1012-2-induced DNA damage among the 3 groups of MAPK pathway.

### 3.5. F1012-2 Induces DNA Damage In Vivo

**Figure 5:**
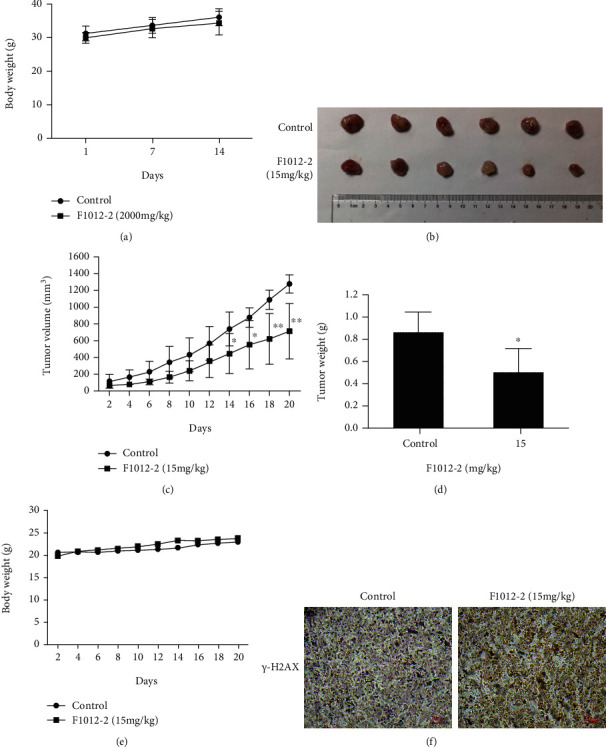
F1012-2 induces DNA damage *in vivo*. (a) The effect of F1012-2 on body weight changes in acute toxicity of mice. (b) The transplanted tumor nude mice were intraperitoneally treated with F1012-2 (15 mg/kg) every other day. After 20 days, the tumor was taken out and photographed. (c) Tumor volume was measured every other day. (d) Tumor weight was measured on day 20. (e) Record the weight of mice with xenografts every other day. (f) Tumor tissues were analyzed for the expression of *γ*-H2AX by immunohistochemistry. Scale bar, 20 *μ*m; magnification, ×10. ^∗^*p* < 0.05 and ^∗∗^*p* < 0.01; *n* = 6.

After 14 days of acute toxicity, maximum dose (2000 mg/kg) did not cause the death of mice, and there was no abnormality in all organs after autopsy. Within 14 days, there was no significant difference in body weight between the control group and the administration group (Figure 5(a)). To investigate F1012-2-induced DNA damage *in vivo*, tumor xenografts were obtained by subcutaneously injecting MDA-MB-231 cells into mice. After 15 mg/kg of F1012-2 treatment for 20 days, the mouse tumors were subjected to immunohistochemical analysis. F1012-2 significantly inhibited tumor growth without toxicity (Figures 5(b)–5(e)). Furthermore, the *γ*-H2AX level in the F1012-2 treatment group was significantly higher than that in the control group detected by immunohistochemistry assay (Figure 5(f)). These data indicated that F1012-2 can inhibit the growth of TNBC cells *in vivo* through DNA damage.

## 4. Discussion

In the present study, we demonstrated that F1012-2, a new sesquiterpene lactone active ingredient extracted from *Eupatorium lindleyanum* DC., significantly induced DNA damage in the Triple-Negative Breast Cancer (TNBC) cell lines. The results showed a better understanding of the anti-TNBC mechanism of F1012-2 is beneficial to the development of novel drugs.

Our previous study has found that F1012-2 has potent antihuman breast cancer activity [11]. In this study, we proved that F1012-2 obviously inhibited cancer cell migration and invasion with wound healing and transwell assay. The comet assay directly demonstrated that DNA strand breaks are an effective way to inhibit the growth of cancer cells. *γ*-H2AX is an indicator of DNA damage in western blotting, immunofluorescence, and immunohistochemistry assays [26, 27]. The results of *γ*-H2AX formation are consistent with those of comet assay. It is well known that higher levels of intracellular ROS will cause oxidative stress, thus inducing DNA damage [28, 29]. As shown in Figure 3, F1012-2 significantly elevated the levels of ROS in MDA-MB-231 and MDA-MB-468 cells.

The MAPK signaling pathway can promote a variety of cellular responses [30]. Many studies have shown that DNA damage caused by the overproduction of ROS can activate ERK, JNK, and p38 signaling pathways that are the three members of MAPK signaling pathway [31]. They can regulate each other and have a significant impact on cell survival and death [32]. As shown in Figure 4, we found that F1012-2 activated the phosphorylation of JNK, ERK, and p38, and the JNK and ERK pathways could be blocked by the corresponding inhibitors. However, compared with F1012-2 alone, pretreatment with p38 inhibitors did not change cell viability and inhibit its phosphorylation, indicating that the JNK and ERK signaling pathways are mainly involved in F1012-2-induced DNA damage and the p38 signaling pathway might play a secondary role.

In conclusion, our findings elucidated that F1012-2 could inhibit the migration and invasion of TNBC cells and had potent induction of DNA damage in the TNBC. A further study showed that F1012-2-induced DNA damage might be triggered by ROS accumulation and involved activation of MAPK pathway, especially the JNK and ERK signaling pathways. These data demonstrate that F1012-2 has an effective anti-TNBC effect and can be used as an alternative chemotherapy agent for TNBC treatment.

## Data Availability

The data used to support the findings of this study are available from the corresponding author upon request.
